# In-stent restenosis and stented-territory infarction after carotid and vertebrobasilar artery stenting

**DOI:** 10.1186/s12883-023-03110-z

**Published:** 2023-02-21

**Authors:** Jae-Chan Ryu, Jae-Han Bae, Sang Hee Ha, Boseong Kwon, Yunsun Song, Deok Hee Lee, Jun Young Chang, Dong-Wha Kang, Sun U. Kwon, Jong S. Kim, Bum Joon Kim

**Affiliations:** 1 Department of Neurology, Asan Medical Center, University of Ulsan College of Medicine, Seoul, Korea; 2 Department of Neurology, Gil Medical Center, Gachon University, Incheon, Korea; 3 Department of Radiology, Asan Medical Center, University of Ulsan College of Medicine, Seoul, Korea; 4 Department of Neurology, Gangneung Asan Hospital, University of Ulsan College of Medicine, Gangneung, Korea

**Keywords:** In-stent restenosis, Ischemic stroke, Carotid stent, Vertebrobasilar stent

## Abstract

**Background:**

Prognosis after vertebrobasilar stenting (VBS) may differ from that after carotid artery stenting (CAS). Here, we directly compared the incidence and predictors of in-stent restenosis and stented-territory infarction after VBS and compared them with those of CAS.

**Methods:**

We enrolled patients who underwent VBS or CAS. Clinical variables and procedure-related factors were obtained. During the 3 years of follow-up, in-stent restenosis and infarction were investigated in each group. In-stent restenosis was defined as reduction in the lumen diameter > 50% compared with that after stenting. Factors associated with the occurrence of in-stent restenosis and stented-territory infarction in VBS and CAS were compared.

**Results:**

Among 417 stent insertions (93 VBS and 324 CAS), there was no statistical difference in in-stent restenosis between VBS and CAS (12.9% vs. 6.8%, *P* = 0.092). However, stented-territory infarction was more frequently observed in VBS than in CAS (22.6% vs. 10.8%; *P* = 0.006), especially a month after stent insertion. HbA1c level, clopidogrel resistance, and multiple stents in VBS and young age in CAS increased the risk of in-stent restenosis. Diabetes (3.82 [1.24–11.7]) and multiple stents (22.4 [2.4–206.4]) were associated with stented-territory infarction in VBS. However, in-stent restenosis (odds ratio: 15.1, 95% confidence interval: 3.17–72.2) was associated with stented-territory infarction in CAS.

**Conclusions:**

Stented-territory infarction occurred more frequently in VBS, especially after the periprocedural period. In-stent restenosis was associated with stented-territory infarction after CAS, but not in VBS. The mechanism of stented-territory infarction after VBS may be different from that after CAS.

**Supplementary Information:**

The online version contains supplementary material available at 10.1186/s12883-023-03110-z.

## Introduction

Atherosclerotic stenosis of the carotid and vertebral arteries are major contributors for ischemic stroke [1]. The most important strategies for preventing ischemic stroke in these arteries are thromboembolism risk reduction and cerebral perfusion improvement. With the development of neuro-interventional devices, carotid artery stenting (CAS) has been widely used as an effective endovascular treatment to achieve this goal [2, 3]. Recently, several studies have demonstrated that not only CAS, but vertebrobasilar artery stenting (VBS) also could be acceptable and safe [4–6].

Vertebrobasilar artery stenosis is associated with a high risk of ischemic stroke; therefore, prediction for recurrent ischemic stroke and a more active treatment strategy after VBS may be needed for arteries with severe stenosis [7, 8]. However, data showing long-term prognosis including in-stent restenosis or ischemic stroke recurrence after VBS are still controversial, and the factors associated with these events are not clearly verified [9–11]. In-stent restenosis is one of the major complications after stent insertion, which was significantly associated with an increased risk of future ischemic stroke after stenting [12, 13]. A considerable proportion of patients show in-stent restenosis after revascularization treatment, and follow-up imaging is performed after stenting to detect in-stent restenosis.

Stroke mechanism and risk factors associated with atherosclerosis differs between ischemic stroke in anterior and posterior circulation [14]. Based on these facts, the incidence and factors associated with in-stent restenosis and stented-territory infarction may differ between VBS and CAS. Here, we investigated the characteristics, long-term incidence, and predictors of in-stent restenosis after VBS and CAS. Moreover, we directly compared the difference in the effect of in-stent restenosis for recurrent infarction between VBS and CAS.

## Methods

### Study population and clinical data

We retrospectively reviewed data of patients who were admitted to the stroke center of our tertiary hospital and received stent insertion at the cervico-cerebral arteries between September 2013 and May 2021. Patients were included if they fulfilled the following criteria: (1) age > 18 years; (2) symptomatic carotid or vertebrobasilar stenosis ≥50% or asymptomatic stenosis ≥70% diagnosed by digital subtraction angiography (DSA). Patients who had never undergone follow-up neuroimaging after stent insertion were excluded.

Baseline demographics and vascular risk factors were collected from all patients. Laboratory values of hemoglobin A1c (HbA1c) and low-density lipoprotein (LDL) cholesterol were obtained during admission, prior to stenting. All patients received aspirin and clopidogrel for at least 5 days before the procedure. The resistance to antiplatelet treatment was tested and expressed as aspirin reaction units (ARUs), P2Y12 reaction units (PRUs), and percentage of platelet inhibition (%PI). ARUs ≥550, PRUs ≥275, or %PI < 20% was defined as resistance to antiplatelet treatment [15]. Regardless of the resistance, all patients received aspirin and clopidogrel during the periprocedural period. Informed consent was not obtained from patients due to the retrospective nature of this study. The local ethics committee approved this study (IRB No. 2022–0348).

### Procedure-related factors

Stenting procedure was performed by neuro-interventionists who were highly experienced in endovascular treatment. The use of catheters, guidewires, and ballooning dilatation was at the discretion of the neuro-interventionist. Wingspan (Boston Scientific), Vision (Abbott Laboratories), and Enterprise (Codman Neuro) stents were used for intracranial stenting, and the Protégé (Covidien), Precise (Cordis), and Acculink (Abbott Laboratories) stents were used for extracranial stenting. The number, location, maximum diameter, and total length of stents, and the ballooning dilatation and pressure were recorded. The degree of stenosis was evaluated according to the North American Symptomatic Carotid Endarterectomy Trial (NASCET) criteria. Stenosis ≥70% was defined as severe and 50–70% as moderate. Symptomatic stenosis was defined as ischemic stroke or transient ischemic attack in a patient within the previous 6 months, resulting from a narrow artery.

### Follow-up neuroimaging and clinical outcomes

All patients were followed up with computed tomography angiography (CTA) and carotid duplex ultrasonography (CDU) within 48 hours after the procedure. After discharge, all patients were followed up with CTA and CDU at 1 month after the procedure. After that, patients were followed up with CTA, CDU, magnetic resonance angiography (MRA), or DSA examination at 6 months after the procedure at the discretion of the stroke neurologist. Thereafter, follow-up neuroimaging was performed every 12 months.

The main outcomes were in-stent restenosis and stented-territory infarction. Restenosis higher than 50% of the residual stenosis just after stent insertion at any period during follow-up was considered as in-stent restenosis, regardless of the neuroimaging modality used. Stented-territory infarction was defined as stented-territory ischemic stroke on diffusion-weighted imaging or transient ischemic attack (TIA), associated with the stented artery. Stented-territory infarction was dichotomized according to the event time (periprocedural [≤1 month] vs. long-term [> 1 month]). We additionally obtained any territory infarction, which was defined as any territory ischemic stroke or TIA regardless of the location of stenting. The events were obtained up to 36 months after the procedure, from the outpatient clinic. All images were analyzed by two stroke neurologists (first author and corresponding author) separately, blinded to all the clinical data.

### Statistical analysis

First, we compared the baseline characteristics, the incidence of in-stent restenosis, and stented-territory infarction between VBS and CAS. The significance of differences was assessed using the Chi-square test, Mann–Whitney U-test, or *t-*test, as appropriate. In-stent restenosis and stented-territory infarction during the follow-up were evaluated using the Kaplan–Meier method. Log-rank tests were used to compare the cumulative incidence between VBS and CAS. In subgroup analysis, for the comparison of intra- vs. extra-cranial stenting, in-stent restenosis and stented-territory infarction during the follow-up period between intra- and extracranial VBS were compared using Kaplan-Meier method and log-rank tests. Moreover, in-stent restenosis and stented-territory infarction between intra- and extracranial CAS were also compared using the same methods.

We performed univariable cox proportional analysis for in-stent restenosis in VBS and CAS separately, and for stented-territory infarction in VBS and CAS. In the latter analysis, in-stent restenosis prior to stented-territory infarction was included as a variable. Variables with potential association (*P* < 0.10) were entered to the multivariable cox proportional analysis. *P*-value < 0.05 was considered statistically significant. The proportional-hazards assumption was checked by examining the Schoenfeld residuals plot and method against time. All analyses were performed using R Software (version 4.0.5; R Foundation for Statistical Computing, Vienna, Austria).

## Results

During the study period, 439 patients received stent insertion at the cervico-cerebral arteries. We excluded 5 (1.1%) patients with stent inserted only in the subclavian artery, and 71 (16.2%) patients without any follow-up neuroimaging after stent insertion. Finally, 363 patients who had undergone 417 stent insertions were included (stenting at more than two arteries: 53 patients). The mean age was 66.7 ± 10.1 years, and 341 (81.8%) patients were males. A total of 324 (77.7%) patients underwent CAS and 93 (22.3%) underwent VBS. The follow-up neuroimaging modalities at the last follow-up were CTA (*n* = 242 [58.3%]), CDU (*n* = 136 [32.6%]), DSA (*n* = 26 [6.2%]), and MRA (*n* = 13 [3.1%]).

### Baseline characteristics

The clinical characteristics of patients who underwent VBS and CAS are summarized in Table 1. The mean age was significantly lower in the VBS group than in the CAS group (63.0 ± 11.8 vs. 67.8 ± 9.3 years, *P* < 0.001). There was less coronary artery disease in the VBS group than in the CAS group (*P* = 0.010). Procedurally, intracranial stenting was more frequently performed in the VBS group than in the CAS group (*P* < 0.001). Moreover, the proportion of symptomatic stenosis before stenting was higher (85 [91.4%] vs. 246 [75.9%], *P* = 0.002), and the maximum diameter and length of stents were smaller and shorter (*P* < 0.001, *P* < 0.001, respectively) in the VBS group than in the CAS group. Furthermore, the proportion of post-ballooning dilatation (24 [25.8%], vs. 150 [46.3%], *P* = 0.001) and maximum balloon pressure (9.5 ± 3.6 atm. vs. 10.7 ± 3.1 atm., *P* = 0.002) was lower in the VBS group than in the CAS group.

**Table 1:** Comparison of baseline characteristics, procedure-related factors, in-stent restenosis and stroke between VBS and CAS

Variable	VBS (*N* = 93)	CAS (*N* = 324)	*P*-value
Age, years	63.0 ± 11.8	67.8 ± 9.3	< 0.001*
Male sex	74 (79.6)	267 (82.4)	0.637
Vascular risk factor			
Hypertension	68 (73.1)	229 (70.7)	0.743
Diabetes	31 (33.3)	130 (40.1)	0.287
Hyperlipidemia	45 (48.4)	157 (48.5)	> 0.999
Coronary artery disease	16 (17.2)	102 (31.5)	0.010*
Stroke history	36 (38.7)	105 (32.4)	0.313
Laboratory finding			
HbA1c, %	6.3 ± 0.9	6.3 ± 1.1	0.843
LDL, mg/dL	89.1 ± 42.8	86.0 ± 34.3	0.529
ARU ≥550	23 (26.4)	60 (19.4)	0.198
PRU ≥275	10 (11.6)	33 (10.5)	0.927
%PI < 20%	44 (51.2)	166 (53.0)	0.852
Procedure-related factors			
Lesion location			< 0.001*
Intracranial	54 (58.1)	28 (8.6)	
Extracranial	39 (41.9)	296 (91.4)	
Symptomatic stenosis	85 (91.4)	246 (75.9)	0.002*
Number of stents ≥2	1 (1.1)	9 (2.8)	0.574
Maximum diameter of stents, mm	4.8 ± 1.8	7.1 ± 1.9	< 0.001*
Total length of stents, mm	25.6 ± 9.6	34.6 ± 10.2	< 0.001*
Pre-ballooning dilatation	79 (84.9)	278 (85.8)	0.968
Post-ballooning dilatation	24 (25.8)	150 (46.3)	0.001*
Maximum balloon pressure, atm	9.5 ± 3.6	10.7 ± 3.1	0.002*
Clinical outcome			
In-stent restenosis	12 (12.9)	22 (6.8)	0.092
Follow-up period, months	20.0 [8.0–36.0]	16.0 [7.0–35.5]	0.203
Any territory infarction	23 (24.7)	43 (13.3)	0.012*
Follow-up period, months	31.0 [11.0–36.0]	24.0 [11.5–36.0]	0.427
Stented-territory infarction	21 (22.6)	35 (10.8)	0.006*
Follow-up period, months	33.0 [11.0–36.0]	24.0 [12.0–36.0]	0.423
Periprocedural (≤1 month)	6 (6.5)	24 (7.4)	0.931
Long-term (> 1 month)	15 (16.1)	11 (3.4)	< 0.001*

Values are expressed as number (%), mean ± standard deviation, and median [interquartile range]

*CAS* Carotid artery stenting, *VBS* Vertebrobasilar artery stenting, *HbA1c* Hemoglobin A1c, *LDL* Low-density lipoprotein, *ARU* Aspirin reaction unit, *PRU* P2Y12 reaction unit, *%PI* Percent platelet inhibition, *TIA* Transient ischemic attack

* Statistically significant (*P* < 0.05)

### In-stent restenosis and stented-territory infarction

For clinical outcomes, in-stent restenosis was observed in 34 (8.2%) stent insertion cases and stented-territory infarction was observed in 56 (13.4%) stent insertion cases during 3 years of follow-up. There was no difference in in-stent restenosis between VBS and CAS groups (12 [12.9%] vs. 22 [6.8%], *P* = 0.092). However, stented-territory infarctions were more frequent in the VBS than in the CAS group (21 [22.6%] vs. 35 [10.8%], *P* = 0.006). Though the occurrence of stented-territory infarction did not differ in the periprocedural period, there was significant difference in long-term stented-territory infarctions between the two groups (15 [16.1%] vs. 11 [3.4%], *P* < 0.001). Any territory infarctions (23 [24.7%] vs. 43 [13.3%], *P* = 0.012) also occurred more frequently in- the VBS than in the CAS group.

**Fig. 1:**
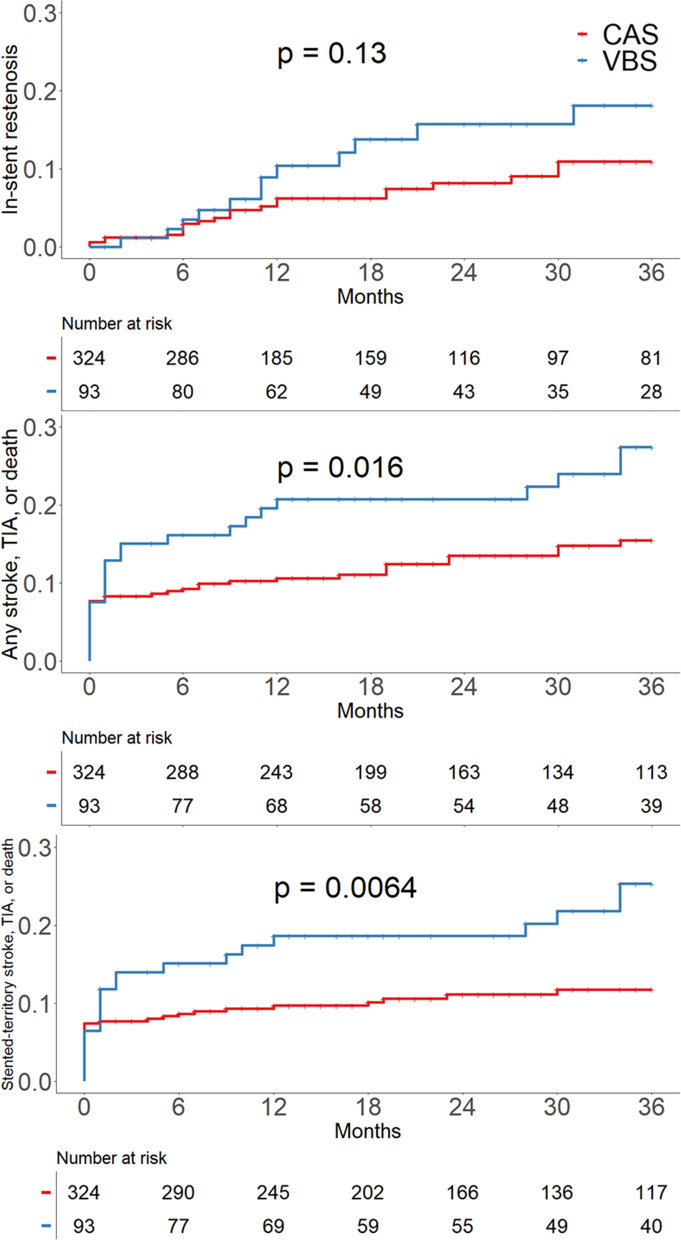
Comparison of in-stent restenosis, any territory infarction, and stented-territory infarction between CAS and VBS. VBS, vertebrobasilar artery stenting; CAS, carotid artery stenting

Kaplan–Meier curve between VBS and CAS is demonstrated in Fig. 1. The incidence of in-stent restenosis in VBS vs. CAS were 10.4% vs. 6.2% at 12 months, 15.7% vs. 8.2% at 24 months, and 18.1% vs. 10.9% at 36 months. There was no significant difference in the occurrence of in-stent restenosis between the two groups (*P* = 0.130). The cumulative incidences of stented-territory infarction in VBS vs. CAS were 18.6% vs. 9.7% at 12 months, 18.6% vs. 11.1% at 24 months, and 25.3% vs. 11.7% at 36 months. The occurrence of any territory infarction and stented-territory infarction were higher in the VBS than in the CAS group (*P* = 0.016, and *P* = 0.006, respectively). In subgroup analysis, there were no statistical differences in the occurrence of in-stent restenosis and stented-territory infarction between intra- and extracranial VBS (*P* = 0.229 and *P* = 0.152). Moreover, the occurrences of in-stent restenosis and stented-territory infarction between intra- and extracranial CAS showed no significant differences (*P* = 0.137 and *P* = 0.215; Supplemental Fig. 1).

### Predictors of in-stent restenosis in VBS and CAS

Multivariable cox proportional analysis showed that HbA1c level (hazard ratio 1.78, 95% confidence interval [1.06–2.98], *P* = 0.029), %PI < 20% (4.20 [1.09–16.2], *P* = 0.037), and number of stents ≥2 (46.3 [2.9–749.0], *P* = 0.007) increased the risk of in-stent restenosis in VBS (Supplemental Table 1).

Univariable cox proportional analysis showed that the age of patients (hazard ratio 0.95, 95% confidence interval 0.91–0.98, *P* = 0.003), number of stents ≥2 (3.51 [0.82–15.0], *P* = 0.091), maximum diameter of stents (0.70 [0.58–0.84], *P* < 0.001), and total length of stents (0.95 [0.92–0.99], *P* = 0.016) were the potential predictors of in-stent restenosis in CAS (Supplemental Table 2). In multivariable analysis, age (0.96 [0.92–1.00], *P* = 0.042) was independently associated with the risk of in-stent restenosis in CAS.

### Predictors of stented-territory infarction in CAS and VBS

Univariable cox proportional analysis showed that diabetes (3.17 [1.09–9.18], *P* = 0.034), HbA1c level (1.48 [0.96–2.28], *P* = 0.075), and the number of stents ≥2 (11.6 [1.42–94.0], *P* = 0.022) were potential predictors for long-term stented-territory infarction in VBS (Table 2). HbA1c levels were not included in the multivariable analysis, as there was multicollinearity between diabetes and HbA1c levels. Diabetes (3.82 [1.24–11.7], *P* = 0.019) and having ≥2 stents (22.4 [2.4–206.4], *P* = 0.006) were associated with long-term stented-territory infarction in VBS.

**Table 2:** Factors associated with long-term stented-territory infarction in VBS

Variable (*N* = 87)	cHR (95% CI)	*P*-value	aHR (95% CI)	*P*-value
Age	1.02 (0.97–1.07)	0.544		
Male sex	1.50 (0.34–6.70)	0.597		
Hypertension	1.40 (0.39–5.02)	0.606		
Diabetes	3.17 (1.09–9.18)	0.034	3.82 (1.24–11.7)	0.019
Hyperlipidemia	0.40 (0.13–1.28)	0.122		
Coronary artery disease	1.91 (0.60–6.09)	0.276		
Stroke history	1.51 (0.53–4.32)	0.438		
HbA1c, %	1.48 (0.96–2.28)	0.075		
LDL, mg/dL	1.00 (0.99–1.01)	0.743		
ARU ≥550	1.33 (0.40–4.43)	0.640		
PRU ≥275	2.03 (0.45–9.15)	0.359		
% PI < 20%	2.23 (0.72–6.90)	0.166		
Intracranial lesion	1.47 (0.49–4.39)	0.491		
Symptomatic stenosis	1.28 (0.17–9.81)	0.810		
Number of stents ≥2	11.6 (1.42–94.0)	0.022	22.4 (2.4–206.4)	0.006
Maximum diameter of stents, mm	0.88 (0.64–1.22)	0.445		
Total length of stents, mm	1.01 (0.96–1.07)	0.676		
Pre-ballooning dilatation	2.36 (0.31–18.0)	0.409		
Post-ballooning dilatation	0.78 (0.22–2.82)	0.708		
Maximum balloon pressure, atm	0.94 (0.81–1.09)	0.388		
In-stent restenosis	2.37 (0.65–8.64)	0.193		

aHR and *P*-value represent the results of multivariable cox proportional analysis. Variables with potential association (*P* < 0.10) were entered to the multivariable cox proportional analysis. Six patients who occurred periprocedural ischemic event were excluded in this analysis

*VBS* Vertebrobasilar artery stenting, *cHR* Crude hazard ratio, *aHR* Adjusted hazard ratio, *HbA1c* Hemoglobin A1c, *LDL* Low-density lipoprotein, *ARU* Aspirin reaction unit, *PRU* P2Y12 reaction unit, *% PI* Percent platelet inhibition

On the other hand, hypertension (0.30 [0.09–1.07], *P* = 0.064), maximum diameter (0.64 [0.48–0.86], *P* = 0.003) and total length of stents (0.93 [0.88–0.98], *P* = 0.009), and in-stent restenosis (24.3 [6.84–86.3], *P* < 0.001) were potentially associated with long-term stented-territory infarction in CAS (Table 3). Among them, in-stent restenosis (15.1 [3.17–72.2], *P* < 0.001) was the predictor of long-term stented-territory infarction in CAS in multivariable cox proportional analysis.

**Table 3:** Factors associated with long-term stented-territory infarction in CAS

Variable (*N* = 300)	cHR (95% CI)	*P*-value	aHR (95% CI)	*P*-value
Age	0.96 (0.91–1.02)	0.163		
Male sex	1.97 (0.25–15.5)	0.521		
Hypertension	0.30 (0.09–1.07)	0.064	0.31 (0.08–1.16)	0.082
Diabetes	1.00 (0.28–3.55)	> 0.999		
Hyperlipidemia	0.13 (0.02–0.99)	0.049	0.22 (0.03–1.86)	0.164
Coronary artery disease	0.85 (0.22–3.28)	0.812		
Stroke history	1.48 (0.42–5.27)	0.541		
HbA1c, %	0.98 (0.56–1.73)	0.949		
LDL, mg/dL	1.00 (0.98–1.02)	0.934		
ARU ≥550	0.49 (0.06–3.88)	0.501		
PRU ≥275	1.99 (0.42–9.36)	0.385		
% PI < 20%	0.90 (0.26–3.12)	0.873		
Intracranial lesion	2.74 (0.58–12.9)	0.203		
Symptomatic stenosis	0.52 (0.15–1.85)	0.313		
Number of stents ≥2	3.19 (0.40–25.2)	0.271		
Maximum diameter of stents, mm	0.64 (0.48–0.86)	0.003	0.95 (0.60–1.53)	0.847
Total length of stents, mm	0.93 (0.88–0.98)	0.009	0.96 (0.89–1.03)	0.276
Pre-ballooning dilatation	0.37 (0.10–1.43)	0.149		
Post-ballooning dilatation	1.35 (0.39–4.68)	0.641		
Maximum balloon pressure, atm	1.03 (0.83–1.29)	0.766		
In-stent restenosis	24.3 (6.84–86.3)	< 0.001	15.1 (3.17–72.2)	< 0.001

aHR and *P*-value represent the results of multivariable cox proportional analysis. Variables with potential association (*P* < 0.10) were entered to the multivariable cox proportional analysis. A total of 24 patients who had a periprocedural ischemic event were excluded from this analysis

*CAS* Carotid artery stenting, *cHR* Crude hazard ratio, *aHR* Adjusted hazard ratio, *HbA1c* Hemoglobin A1c, *LDL* Low-density lipoprotein, *ARU* Aspirin reaction unit, *PRU* P2Y12 reaction unit, *% PI* Percent platelet inhibition

## Discussion

Our study shows that stented-territory infarctions were significantly more frequent after VBS than after CAS, especially in the long-term. The predictors for in-stent restenosis and stented-territory infarction also differed; in-stent restenosis was a predictor for stented-territory infarction for CAS, but not for VBS. For those who received VBS, in-stent restenosis was associated with HbA1c level, clopidogrel resistance, and multiple stents; while stented-territory infarction was associated with diabetes and multiple stents.

The cumulative incidences of in-stent restenosis reported from separate studies on VBS and CAS, including a meta-analysis, were in line with our current results [13, 16–19]. Furthermore, previous meta-analysis showed the indifference of the occurrence of in-stent restenosis between intra- and extracranial VBS [9]. Although the results of previous reports were heterogenous, the periprocedural complication rate was low and the safety was acceptable for both intra-and extra-cranial VBS [9–11, 20, 21]. In the current study, periprocedural complication rate was similar between VBS and CAS. However, long-term stented-territory infarction was more frequently observed in VBS than in CAS. This may be explained by the difference in the location of stenting and stroke mechanism. In our patient cohort, half of the patients received stenting at the origin of the vertebral artery, and the other half received stenting at the distal vertebral or basilar artery. Those who received stenting at the distal vertebral or basilar artery might have had a stroke due to occlusion of the perforators. The occlusion of the perforators can be caused by in-stent atherosclerosis, even with stenosis less than 50% [14]. Therefore, though the incidence of in-stent restenosis was not significantly higher in those who had received VBS, still the risk of stroke may be higher in VBS. During the long-term follow-up period, a considerable portion of patients who had undergone VBS had stroke due to occlusion of the perforators (Supplemental Table 3). However, there was no significant difference in the proportion of long-term stented-territory ischemic event between the intra- and extra-cranial VBS (10/54 [18.5%] vs. 5/39 [12.8%], respectively, *P* = 0.461; data not shown). A study with larger number of patients comparing stented-territory infarction between intra- and extracranial VBS may be needed to clarify this issue.

Stented-territory infarction after VBS was not associated with in-stent restenosis. On the contrary, stented-territory infarction after CAS, which is mostly caused by artery-to-artery embolism, was significantly associated with in-stent restenosis [13, 22]. Stenosis degree more than 50% is critical for platelet aggregation and distal embolism. Younger age increased the risk of in-stent restenosis in CAS. Lower intimal cell proliferation and consequent lower intimal hyperplasia could help explaining this result [23]. In case of VBS, in-stent restenosis and stented-territory infarction was more associated with metabolic components, such as high HbA1c levels or the history of diabetes. Diabetes and metabolic syndrome are well-known risk factors for stroke in the posterior circulation [24]. Diabetes is also a major risk factor for branch atheromatous disease, which may be an important stroke mechanism in those who had received VBS [25]. Actively controlling diabetes, which may prevent development of atherosclerosis within the stent placed at the posterior circulation may be important after VBS.

Our study has some limitations. First, due to the retrospective nature of this study, patients with follow-up neuroimaging were limited, which might have caused a selection bias. Second, follow-up neuroimaging modality was heterogenous. This heterogenicity might have caused discrepancies in the incidence of in-stent restenosis, subsequently affecting the results. Third, we alternatively investigated the number, diameter, and length of stents, instead of the arterial diameter, the ratio of stenosis, and the maximum size of balloon. This difference might have affected the results of our study. Finally, this study was conducted at a single center; therefore, it is difficult to generalize the results. However, owing to this, the procedure was standardized, and it may not have affected the result.

Despite these limitations, we directly compared the prevalence and risk factors associated with VBS and CAS. The periprocedural risk was similar, however, VBS showed a higher risk of stented-territory infarction at long-term follow-up. In-stent restenosis was a risk factor for stented-territory infarction in CAS, whereas diabetes was associated with in-stent restenosis and stented-territory infarction in VBS.

## Supplementary Information

**Additional file 1: Supplemental Table 1.** Hazard ratio for in-stent restenosis using a cox proportional analysis in VBS. **Supplemental Table 2.** Hazard ratio for in-stent restenosis using a cox proportional analysis in CAS. **Supplemental Table 3.** Stroke mechanism of stented-territory infarction in VBS. **Supplemental Figure 1.** A) Comparison of in-stent restenosis and stented-territory infarction between intra- and extracranial VBS. B) Comparison of in-stent restenosis and stented-territory infarction between intra- and extracranial CAS.

## Data Availability

Data are available upon reasonable request to the corresponding author.

## Abbreviations

CAS: Carotid artery stenting
VBS: Vertebrobasilar artery stenting
DSA: Digital subtraction angiography
HbA1c: Hemoglobin A1c
LDL: Low-density lipoprotein
ARUs: Aspirin reaction units
PRUs: P2Y12 reaction units
%PI: Percentage of platelet inhibition
NASCET: North American Symptomatic Carotid Endarterectomy Trial
CTA: Computed tomography angiography
CDU: Carotid duplex ultrasonography
TIA: Transient ischemic attack
