# Supplementary material for: In-stent restenosis and stented-territory infarction after carotid and vertebrobasilar artery stenting
Source: BMC Neurol. 2023 Feb 21;23:79. doi: 10.1186/s12883-023-03110-z (PMC9942307; doi:10.1186/s12883-023-03110-z)

Supplemental Table 1. Hazard ratio for in-stent restenosis using a cox proportional analysis in VBS.

| Variable (N=93) | cHR (95% CI) | P-value | aHR (95% CI) | P-value |
| --- | --- | --- | --- | --- |
| Age | 1.01 (0.96–1.05) | 0.823 |  |  |
| Male sex | 1.26 (0.28–5.78) | 0.763 |  |  |
| Hypertension | 1.78 (0.39–8.12) | 0.458 |  |  |
| Diabetes | 1.54 (0.49–4.85) | 0.462 |  |  |
| Hyperlipidemia | 0.98 (0.31–3.03) | 0.968 |  |  |
| Coronary artery disease | 1.02 (0.22–4.69) | 0.980 |  |  |
| Stroke history | 0.82 (0.25–2.74) | 0.750 |  |  |
| HbA1c, % | 1.65 (1.04–2.64) | 0.036 | 1.78 (1.06–2.98) | 0.029 |
| LDL, mg/dL | 0.99 (0.98–1.01) | 0.372 |  |  |
| ARU ≥550 | 0.57 (0.12–2.60) | 0.467 |  |  |
| PRU ≥275 | 0.88 (0.11–6.84) | 0.900 |  |  |
| %PI <20% | 3.98 (1.07–14.8) | 0.039 | 4.20 (1.09–16.2) | 0.037 |
| Intracranial lesion | 2.19 (0.59–8.10) | 0.240 |  |  |
| Symptomatic stenosis | 0.93 (0.12–7.24) | 0.944 |  |  |
| Number of stents ≥2 | 86.5 (5.0–1383) | 0.002 | 46.3 (2.9–749.0) | 0.007 |
| Maximum diameter of  stents, mm | 0.88 (0.63–1.24) | 0.466 |  |  |
| Total length of stents, mm | 1.02 (0.96–1.08) | 0.601 |  |  |
| Pre-ballooning dilatation | 1.94 (0.25–15.0) | 0.528 |  |  |
| Post-ballooning dilatation | 2.21 (0.70–6.97) | 0.177 |  |  |
| Maximum balloon pressure, atm | 1.13 (0.96–1.33) | 0.145 |  |  |

Adjusted HR and P-value represent the results of multivariable cox proportional analysis. Variables with potential association (P <0.10) were entered to the multivariable cox proportional analysis.

VBS, vertebrobasilar artery stenting; cHR, crude hazard ratio; aHR, adjusted hazard ratio; HbA1c, hemoglobin A1c; LDL, low-density lipoprotein; ARU, aspirin reaction unit; PRU, P2Y12 reaction unit; %PI, percent platelet inhibition.

Supplemental Table 2. Hazard ratio for in-stent restenosis using a cox proportional analysis in CAS.

| Variable (N=324) | cHR (95% CI) | P-value | aHR (95% CI) | P-value |
| --- | --- | --- | --- | --- |
| Age | 0.95 (0.91–0.98) | 0.003 | 0.96 (0.92–1.00) | 0.042 |
| Male sex | 1.03 (0.35–3.05) | 0.957 |  |  |
| Hypertension | 0.99 (0.40–2.43) | 0.980 |  |  |
| Diabetes | 1.50 (0.65–3.45) | 0.345 |  |  |
| Hyperlipidemia | 0.77 (0.33–1.79) | 0.539 |  |  |
| Coronary artery disease | 1.90 (0.82–4.39) | 0.136 |  |  |
| Stroke history | 1.63 (0.69–3.82) | 0.263 |  |  |
| HbA1c, % | 1.15 (0.82–1.61) | 0.422 |  |  |
| LDL, mg/dL | 0.99 (0.98–1.01) | 0.280 |  |  |
| ARU ≥550 | 1.03 (0.35–3.06) | 0.953 |  |  |
| PRU ≥275 | 0.40 (0.05–2.99) | 0.374 |  |  |
| %PI <20% | 0.83 (0.35–1.95) | 0.666 |  |  |
| Intracranial lesion | 2.23 (0.75–6.29) | 0.148 |  |  |
| Symptomatic stenosis | 2.13 (0.63–7.21) | 0.223 |  |  |
| Number of stents ≥2 | 3.51 (0.82–15.0) | 0.091 | 3.49 (0.52–23.4) | 0.199 |
| Maximum diameter of  stents, mm | 0.70 (0.58–0.84) | <0.001 | 0.73 (0.53–1.02) | 0.061 |
| Total length of stents, mm | 0.95 (0.92–0.99) | 0.016 | 1.00 (0.94–1.06) | 0.951 |
| Pre-ballooning dilatation | 1.71 (0.40–7.31) | 0.471 |  |  |
| Post-ballooning dilatation | 1.08 (0.46–2.55) | 0.862 |  |  |
| Maximum balloon pressure, atm | 1.03 (0.89–1.19) | 0.674 |  |  |

Adjusted HR and P-value represent the results of multivariable cox proportional analysis. Variables with potential association (P <0.10) were entered to the multivariable cox proportional analysis.

CAS, carotid artery stenting; cHR, crude hazard ratio; aHR, adjusted hazard ratio; HbA1c, hemoglobin A1c; LDL, low-density lipoprotein; ARU, aspirin reaction unit; PRU, P2Y12 reaction unit; % PI, percent platelet inhibition.

Supplemental Table 3. Stroke mechanism of stented-territory infarction in VBS.

| Stented-territory infarction | Number of patients (N=21) |
| --- | --- |
| Periprocedural stented-territory infarction | 6 (28.6) |
| Artery-to-artery embolism | 3 |
| Branch atheromatous disease | 2 |
| TIA | 1 |
| Long-term stented-territory infarction | 15 (71.4) |
| Artery-to-artery embolism | 5 |
| Branch atheromatous disease | 4 |
| TIA | 6 |

Values are expressed as numbers (%).

VBS, vertebrobasilar stenting; TIA, transient ischemic attack.

Supplemental Figure 1. A) Comparison of in-stent restenosis and stented-territory infarction between intra- and extracranial VBS. B) Comparison of in-stent restenosis and stented-territory infarction between intra- and extracranial CAS.

VBS, vertebrobasilar artery stenting; CAS, carotid artery stenting


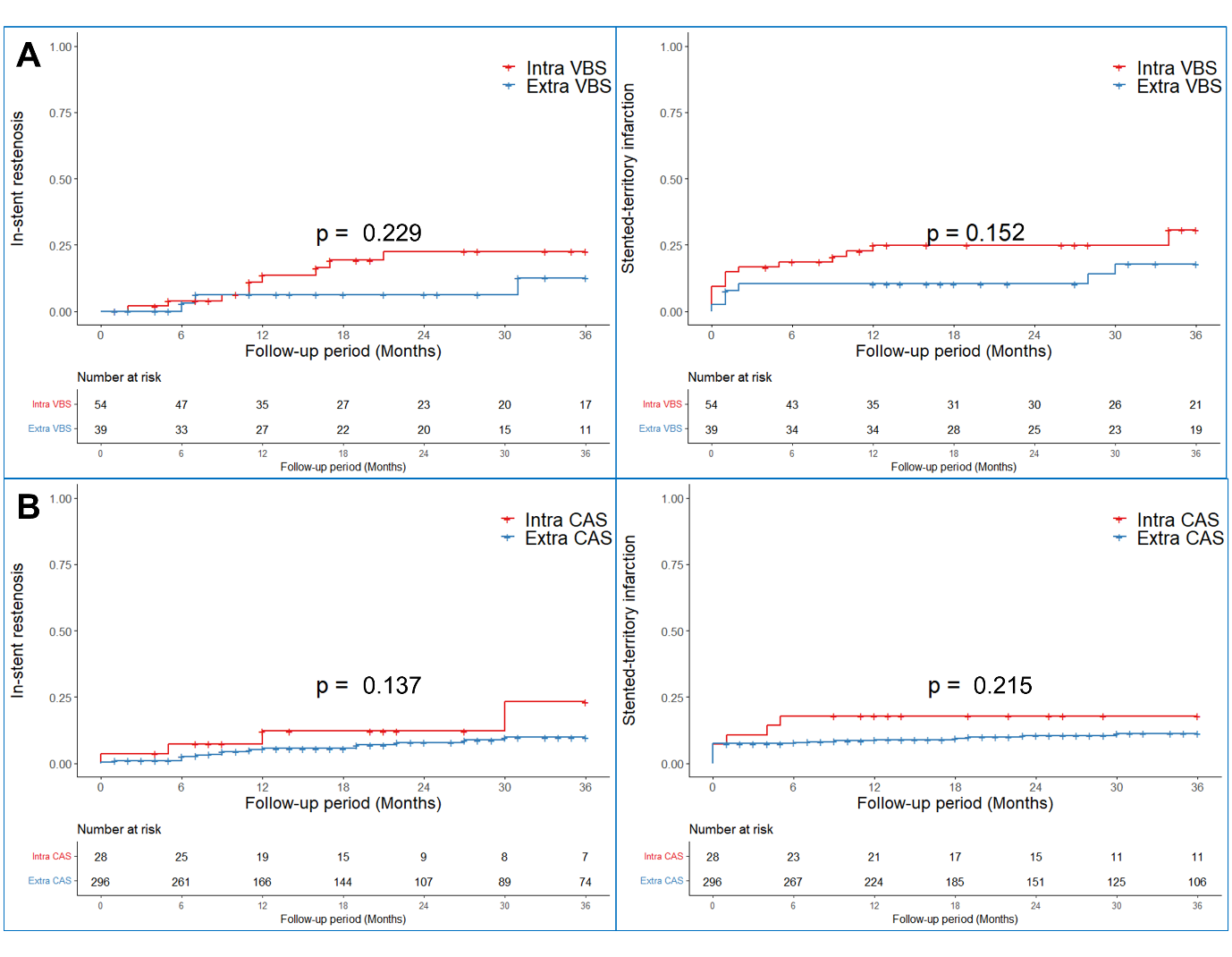

Supplement: Supplementary file 1 — Additional file 1: Supplemental Table 1. Hazard ratio for in-stent restenosis using a cox proportional analysis in VBS. Supplemental Table 2. Hazard ratio for in-stent restenosis using a cox proportional analysis in CAS. Supplemental Table 3. Stroke mechanism of stented-territory infarction in VBS. Supplemental Figure 1. A) Comparison of in-stent restenosis and stented-territory infarction between intra- and extracranial VBS. B) Comparison of in-stent restenosis and stented-territory infarction between intra- and extracranial CAS. [file 12883_2023_3110_MOESM1_ESM.docx]
